# Sarcoidosis: Pitfalls and Challenging Mimickers

**DOI:** 10.3389/fmed.2020.594275

**Published:** 2021-01-11

**Authors:** Naureen Narula, Michael Iannuzzi

**Affiliations:** Staten Island University Hospital, New York, NY, United States

**Keywords:** sarcoidosis, cardiac sarcoidosis (CS), neurosarcoidosis, hypercalcemia (HCM), hypersentivity pneumonitis, sarcoidosis associated pulmonary hypertension, aseptic meningitis

## Abstract

Sarcoidosis, a systemic granulomatous disease of unknown etiology, may mimic other conditions at presentation often resulting in delayed diagnosis. These conditions include infections, neoplasms, autoimmune, cardiovascular, and drug-induced diseases. This review highlights the most common sarcoidosis mimics that often lead to pitfalls in diagnosis and delay in appropriate treatment. Prior to invasive testing and initiating immunosuppressants (commonly corticosteroids), it is important to exclude sarcoid mimickers.

## Introduction

Jonathon Hutchinson, an English physician, first described sarcoidosis in 1877 (1). Over the last few decades, substantial progress has been made to better define the clinical, radiological, immunological, and pathological features of sarcoidosis. The diagnosis of sarcoidosis is based on three major criteria: clinical presentation compatible with sarcoidosis, presence of non-necrotizing granulomatous inflammation in one or more tissue samples, and the exclusion of alternative causes of granulomatous disease. The American Thoracic Society recently published an official clinical practice guideline in which a panel of experts discuss and summarize evidence-based diagnosis of sarcoidosis (2). Despite advancements clinicians often remain challenged in reaching a diagnosis of sarcoidosis.

### Infections

Nearly all infectious causes of granulomas may present similarly to sarcoidosis (Table 1) (3–7) thus excluding an infectious etiology must be routine and relies more on laboratory studies rather than differentiating clinical features. Table 1 highlights the recommended laboratory studies to be considered during the workup of a patient. Sarcoidosis is an often overlooked cause of fever of unknown origin (FUO) (8). About one-third of patients with sarcoidosis exhibit non-specific constitutional findings such as fever, lethargy, night sweats, and weight loss.

**Table 1:** Infectious causes of granulomas and the laboratory studies to consider to rule out infection.

**1. Bacteria**
Actinomyces Bartonella *Borrelia burgdorferi* Brucellosis *Mycobacterium tuberculosis* Non-tuberculous mycobacterium Nocardia Q fever *Treponema pallidum* Whipples disease
**2. Fungi**
Aspergillosis Blastomycosis Candida Cryptococcus Histoplasma
**3. Parasitic**
Leshmaniosis Schistosomiasis
**4. Viral**
Cytomegalovirus Epstein barr
**Laboratory studies to rule out infection:**
1. Histopathologic examination
Ziehl–Neelsen stain or Auramine–rhodamine fluorescence for mycobacteria Grocott methenamine silver stain for fungi TB PCR
2. Culture for bacteria (aerobic and anaerobic), fungi, mycobacteria
3. Serology for histoplasma, syphilis, lyme disease

In developing countries with high TB burdens, diagnosing sarcoidosis can be particularly challenging. TB is classically characterized by caseating granulomas whereas sarcoidosis has non-caseating epithelioid cell granulomas. However, when caseous necrosis is not seen and acid-fast staining of biopsy specimens is negative then a patient with suspected TB infection can be mistakenly diagnosed with sarcoidosis (3). In an endemic area, clinical judgment is crucial. Radiological findings of upper lobe predominant cavitary lesions favor the diagnosis of TB, as cavity formation occurs in only 3% of sarcoidosis cases (9).

Endobronchial ultrasound-guided transbronchial needle aspiration (EBUS-TBNA) to obtain TB-PCR samples makes ruling out TB more certain. In a study by Eom et al., 86 specimens were examined in 46 patients and the sensitivity, specificity, positive predictive value, negative predictive value, and diagnostic accuracy were analyzed. EBUS-TBNA TB PCR was found to be 56%, 100%, for sensitivity and specificity, respectively. Positive and negative predictive values were 100%, and 81%. Diagnostic accuracy was 85% (10). Characterization of sonographic features of lymph nodes by an EBUS can also aid in differentiating TB associated lymphadenopathy (LAD) from sarcoidosis. Dhooria et al. analyzed 250 EBUS-guided TBNA procedures in patients with intrathoracic LAD and found that sonographic features of heterogeneous echotexture and coagulation necrosis are suggestive of TB rather than sarcoidosis. A combination of a positive tuberculin skin test (TST) and either heterogeneous echotexture or coagulation necrosis sign had a specificity of 98% and a positive predictive value of 91% for a diagnosis of tuberculosis (11).

Heterogeneous echotexture (53.4 vs. 12.6%, *P* < 0.001) and coagulation necrosis (26.1 vs. 3.3%; *P* < 0.001) are suggestive of TB rather than sarcoidosis. A combination of a positive tuberculin skin test (TST) and either heterogeneous echotexture or coagulation necrosis sign had a specificity of 98% and positive predictive value of 91% for a diagnosis of tuberculosis (11). Use of an interferon-γ (IFN-γ) release assay has been reported to demonstrate a better predictive ability than tuberculin skin tests (12). Culture although time-consuming is still considered as a gold standard test for the diagnosis of Tuberculosis (13).

An accurate and timely diagnosis of sarcoidosis helps prevent unnecessary antituberculosis therapy (ATT) drug exposure. An accurate diagnosis of TB prevents exposure to immunosuppressive agents. Concomitant tuberculosis and sarcoidosis is rare (14).

The presence of exudative pleural effusions may favor other diagnoses. Pleural effusions associated with sarcoidosis are uncommon (8.2%) and can be present at the time of diagnosis or at a later time, coinciding with an exacerbation (15, 16). These effusions are typically right sided, exudative, and lymphocytic predominant. Eosinophilic and neutrophilic effusions have been reported, but are less common. Pleural fluid in sarcoidosis is characterized by having a high CD4/CD8 ratio and frequently sarcoid related pleural effusions resolve spontaneously but still may require treatment with corticosteroids (17).

The presence of an exudative effusion in the setting of sarcoidosis warrants infectious workup e.g., parapneumonic effusion, or empyema. Patients on immunosuppressive agents are particularly susceptible to opportunistic infections (18–20). The failure of pleural effusions to respond to corticosteroid treatment should raise suspicion for an underlying opportunistic infection or other complications such as pulmonary embolism. Table 2 highlights the characteristic differentiating features in patients with common infectious etiologies which can mimic sarcoidosis.

**Table 2:** Highlights the characteristic differentiating features in patients with common infectious etiologies which can mimic sarcoidosis.

	**Features**	**Sarcoidosis**	***Mycobacterium tuberculosis***	**Histoplasmosis**
A	**Lab findings** 1. ACE level 2. Abnormal calcium metabolism 3. Kveim-Siltzbach test 4. Mantoux test 5. Other diagnostic tests	Elevated ACE (50–80%) (19–21) Elevated vitamin D Hypercalcemia Hypercalciuria (22) Positive in 60% (23) Anergic; Negative in 90% (24)	Elevated ACE in 9% (22, 25) Hypovitaminosis D Hypercalcemia rare but can be seen in MTB IRD cases (26) Negative Positive in 65–94% (27, 28) PCR MTB	Elevated in 25% (29, 30) Hypercalcemia rare but can be seen (31) Negative Negative Histoplasma antigen in urine (sensitivity 93–100%) (Gold standard test) and serum (32) - PCR assays for the Hcp 100 gene - Histoplasmin sensitivity skin test
B	BAL findings	- CD4/CD8 ratio >3.5 (33) - Elevated ACE (34, 35)	BAL AFB smear (sensitivity 38.1%) BAL culture (sensitivity 74.5%) (35, 36) Elevated CD4^+^/CD8^+^ ratio (37)	Histoplasmosis Antigen in BAL (38)
C	Histopathology	Non-caseating granulomas	Caseating granulomas - AFB^+^ bacilli - Isolation of MTB	-Non-caseating granulomas - Isolation of *H. capsulatum* from tissue specimens (gold standard) - methenamine silver or PAS stains revealing narrow-based budding yeasts
D	1. Radiological Hilar and mediastinal LAD 2. EBUS	Bilateral Hilar LAD when present is symmetrical Nodules Reticulonodular opacities Cavitation rare Granular appearance in lymph nodes -highest specificity (99.3%) for the diagnosis of sarcoidosis (39).	Asymmetrical, unilateral Upper-lobe infiltrates with cavitation, tree-in-bud, macro-nodular infiltrates (3). Cavitation is more common (9) Heterogeneous echotexture (53.4% *P* < 0.001) and coagulation necrosis (26.1% *P* < 0.001) (11)	Asymmetrical, unilateral (40) Fibrosing mediastinitis Reticulonodular opacities Cavitation is rare (41) Non-specific
E	Treatment	Immunosuppressant like corticosteroids	ATT	Itraconazole, AMB Streptomycin

*AFB, Acid Fast Bacilli; ATT, Anti-Tuberculosis Treatment; AMB, Amphotericin B; MTB, Mycobacterium tuberculosis; LAD, Lymphadenopathy; PCR, Polymerase Chain Reaction; MTB-IRD, Mycobacterium tuberculosis Immune Reconstitution Disease*.

## Granulomatous Chronic Interstitial Lung Disease

Along with sarcoidosis, granuloma formation is a feature of other chronic interstitial lung diseases such as chronic beryllium disease (CBD) and hypersensitivity pneumonitis (HP).

### Chronic Beryllium Disease (CBD)

Chronic beryllium disease (CBD) is clinically and pathologically indistinguishable from sarcoidosis. CBD risk is associated with fluorescent light manufacturing and in industries such as nuclear energy, ceramics, aerospace, automotive, electronics, and telecommunications (42). Non-occupational exposure to CBD has been reported in family members of factory workers likely due to second-hand exposure and in residents of communities near the beryllium factories.

Symptoms of CBD include dry cough, progressive dyspnea on exertion, fatigue, and night sweats. The radiological features vary with common chest CT findings including small nodules, ground-glass opacification, mild hilar adenopathy, and septal lines. A diagnosis of CBD is favored over sarcoidosis when there is documented occupational exposure to beryllium. Beryllium exposure should be considered at the time of sarcoidosis diagnosis as well as in chronic corticosteroid-resistant sarcoidosis to ensure they are removed from ongoing exposure to immunosuppressants.

A diagnosis of CBD can be aided by the following three criteria: (1) symptomatic disease with a histopathological demonstration of non-caseating granuloma, pulmonary function impairment, and abnormal chest radiographs; (2) proof of beryllium sensitization by two independently positive beryllium lymphocyte proliferation assays (BeLPTs) in the absence of treatment with systemic corticosteroids for preceding 3 months, and (3) proof of beryllium exposure (43, 44). Beryllium sensitization is diagnosed when either criteria 2 or 3 are fulfilled without criterium 1.

Management of suspected CBD involves stopping all further exposure and continued clinical surveillance. Therapy with corticosteroids may be beneficial in selected patients, but the response is variable. Oral prednisone (doses ranging from 20 to 40 mg/day) for 3–6 months followed by a gradual taper to the lowest effective dose is deemed acceptable. Unfortunately, CBD is typically a progressive disease and lifelong treatment may be required. BeLPT should be offered to any patient with sarcoidosis who has worked around metal-dust or fumes (45).

### Hypersensitivity Pneumonitis

Hypersensitivity pneumonitis (HP) has clinical, radiological, and histopathological features that overlap with sarcoidosis. HP pathogenesis results from a combination of immune complex-mediated (type III) and delayed-type (type IV) hypersensitivity reactions to antigen inhalation in a susceptible person. Cryptogenic HP is a subtype of HP in which despite typical HP features an inciting exposure is not identified (46–48).

Table 3 illustrates comparative features between hypersensitivity pneumonitis and sarcoidosis.

**Table 3:** Compares the characteristics of CBD with sarcoidosis across various clinical characteristics, lab abnormalities, and imaging studies.

	**Sarcoidosis**	**Hypersensitivity pneumonitis (HP)**	**CBD**
Exposure history	Unknown	May have a history of an allergen exposure	Exposure to beryllium; Occupational history
Age of diagnosis	Pea peak incidence at age of 20–39 years (49)	The median age of diagnosis 65 years	Mean age at time of diagnosis of CBD 43.9 (25–80) years (50)
Laboratory	ACE level elevated (>80%)	Serum IgG to potential antigens associated with HP (sensitivity and specificity of 83 and 68%) (48)	ACE may be elevated in 22% case (44, 51). BeLPT: abnormal (peripheral blood and/or BAL) (52) Patch testing (BeSO_4_ or BeF): abnormal (peripheral blood and/or BAL)
Radiological	- Symmetrical and bilateral Hilar LAD - Nodules and/or Reticulonodular opacities	Features depend on subtype- non-fibrotic or fibrotic HP - GGO, mosaic attenuation, centrilobular nodules, fibrosis (irregular linear opacities; traction bronchiectasis and honeycombing) - Fibrosis is most severe in the mid or mid and lower lung zones or equally distributed in the three lung zones with relative basal sparing. Head cheese sign/three-density pattern (47)	The most common radiographic abnormalities include diffuse small round and reticular opacities. Hilar adenopathy, linear scars, lung distortion, bullae, and pleural thickening are found less commonly associated (53)
Histopathology	Well-formed, non-necrotizing granulomas, showing a lymphangitic distribution.	Small and poorly formed granulomas, comprising loose, poorly circumscribed clusters of epithelioid and multinucleated cells (54). Bronchiolocentric inflammation In Fibrotic HP-subpleural and centriacinar fibrosis, with or without bridging fibrosis. May have features that overlap with a UIP pattern (55)	Well-formed, non-necrotizing granulomas, and/or mononuclear cell interstitial cell infiltrates on endobronchial or transbronchial biopsy Calcific inclusions reported
BALF	Lymphocytic CD4^+^:CD8^+^ ratio >3.5	Lymphocytic (>30% for non- and ex-smokers and >20% for current smokers) (56) Low CD4^+^/CD8^+^ ratio (mean values of 0.5–1.5) Increased expression of CD80/CD86 (48, 57)	Lymphocytosis 41–53% (44, 58) BeLPT BAL
Spontaneous resolution	Depends upon the stage	Rare	Rare
Treatment	Immunosuppressants (corticosteroids)	Removal from exposure - Corticosteroids (59)	Removal from further exposure. In symptomatic cases, corticosteroids/immunosuppressants

*ACE, angiotensin converting enzyme; BALF, bronchoalveolar fluid; BeLPT, beryllium lymphocyte proliferation assay; CBD, chronic beryllium disease*.

HP can be categorized by disease duration into acute, subacute, and chronic subtypes (60). Raghu et al. recently proposed categorization based on the presence or absence of radiological and/or histopathological fibrosis into either fibrotic HP or non-fibrotic HP, which has been widely accepted (61).

The median age at diagnosis is 65 years which is somewhat older than what is typical of sarcoidosis (20–39 years). Common presenting symptoms of HP include dyspnea, cough, wheezing, and, less frequently, constitutional symptoms such as weight loss. Physical examination may reveal the presence of rales. An acute presentation is more consistent with the non-fibrotic subtype and an insidious presentation is more consistent with fibrotic HP (47, 62, 63).

The radiological features of HP depend upon the subtype. Typical radiologic features of non-fibrotic HP include high-resolution CT (HRCT) showing parenchymal infiltration with ground-glass opacities (GGO), and mosaic attenuation. HRCT may also demonstrate small airway disease, which can be described as ill-defined, small (<5 mm) centrilobular nodules on inspiratory images and air trapping on expiratory images, both in a diffuse distribution. Typical radiological features associated with fibrotic HP include an HRCT pattern of irregular linear opacities and or/coarse reticulation with lung distortion; traction bronchiectasis and honeycombing, all typical of lung fibrosis, and at least one abnormality that indicates small airway disease. The presence of three different lung densities, previously referred by radiologists as “head-cheese sign” and recently being referred to as the “three-density pattern,” is characteristically associated with HP (61). Radiologically, granulomas associated with HP are interstitial and do not follow the lymphatics as in sarcoid. Also, sarcoidosis normally spares the lung bases. For both HP and advanced sarcoidosis, a prognosis-predicting factor is the extent of the fibrotic score calculated on HRCT scans (64, 65). Further, HRCT can help differentiate active inflammation from fibrosis in patients with advanced-stage sarcoidosis (66). Honeycombing is uncommon in sarcoidosis but if present usually involves the middle and upper lung zones with relative sparing of the lung bases. The presence of lower lung honeycombing would make it difficult to distinguish between HP, sarcoidosis, and idiopathic pulmonary fibrosis (59).

Bronchoalveolar lavage fluid (BALF) analysis of patients with HP reveals a predominance of lymphocytes, like sarcoidosis. A meta-analysis of 53 studies (3,112 patients) by Raghu et al. demonstrated that patients with HP had a higher proportion of BALF lymphocytes than patients with sarcoidosis (MD, 19%; 95% CI, 17–21%). This was found regardless of whether the study enrolled patients with non-fibrotic HP (17 studies; MD, 25%; 95% CI, 22–27%), fibrotic HP (16 studies; MD, 16%; 95% CI, 11–20%), or mixed populations with both non-fibrotic and fibrotic HP (21 studies; MD, 18%; 95% CI, 15–20%). For distinguishing non-fibrotic HP from sarcoidosis, BALF lymphocyte thresholds of 20, 30, and 40% yielded decreased sensitivities for HP of 95, 88, and 76%, respectively, but increased specificities of 26, 43, and 61%, respectively, with an area under the curve of 0.71 (95% CI, 0.67–0.74) (61).

A diagnosis of HP requires:

1. The presence of an exposure, defined as a positive exposure history, serum IgG testing against potential antigens associated with HP, and/or specific inhalation challenge
2. BALF revealing lymphocytosis or histopathological findings on biopsy
3. Typical radiographic features as described above (GGO and mosaic attenuation for non-fibrotic HP and fibrotic changes with small airway involvement for fibrotic HP).

A diagnosis of HP can be made with high confidence in patients with an identified provocative exposure and who have a typical HP radiological pattern on HRCT and with BALF revealing predominant lymphocytosis. For less straightforward cases, a multidisciplinary team consisting of a pulmonologist experienced in interstitial lung disease (ILD), a chest radiologist, and when necessary, a pathologist familiar with histopathological features of ILD and HP.

Treatment involves prompt and complete avoidance of further exposure to the inducer. Systemic corticosteroids are used in treatment however, their use has no effect on long-term outcomes and is often reserved for patients with more severe symptoms (67, 68). Fibrotic HP is associated with worse prognosis particularly with persistent exposure to the inciting agent, cigarette smoking, lower baseline vital capacity, and lack of BALF lymphocytosis (49, 62, 69, 70). Patients with the progressive disease should be evaluated for lung transplantation (55).

## Thrombosis; Pulmonary Hypertension

When presented with a patient with sarcoidosis and worsening shortness of breath, an assessment for pulmonary embolism (PE) and pulmonary hypertension (PH) is warranted. PE and sarcoidosis associated pulmonary hypertension (SAPH) may often be misdiagnosed as an exacerbation of sarcoidosis.

## Venous Thromboembolism (VTE) Associated With Sarcoidosis

Sarcoidosis, as with other chronic inflammatory conditions, has been associated with an increased risk of venous thromboembolism (VTE) (71–73). Swigris et al. reported a greater than 2-fold higher risk of pulmonary embolism in patients with sarcoidosis when compared to the general population (74). In addition to PE, thrombosis secondary to localized inflammation has also been reported in the literature. Mural thrombosis in myocardial sarcoidosis, cerebral vein thrombosis in neurosarcoidosis and thoracic vein thrombosis in mediastinal sarcoidosis have all been reported (75–77). While the precise mechanism is not yet defined, chronic inflammation associated with sarcoidosis likely predisposes to endothelial cell injury with the inflammatory cytokines activating the coagulation cascade (78–80). Extrinsic vascular compression at a mediastinal or hilar level may lead to venous stasis thus progressing to localized thrombosis (77). Up to 38% of sarcoidosis patients demonstrate the presence of antiphospholipid antibodies (81). The use of glucocorticoids further increases the risk of VTE (82).

## Sarcoidosis-Associated Pulmonary Hypertension (SAPH)

Fischer et al. reported elevated pulmonary-artery pressure in 6–23% of patients at rest and in as many as 43% with exertion (83). SAPH has been reported in up to 74% of patients with advanced sarcoidosis (84, 85). In one case-series, Schorr et al. reported a 7-fold increased risk for death over a 3-year follow-up in patients with SAPH (86). Pulmonary fibrosis leading to obliteration of the pulmonary vessels is considered the most common mechanism for developing PH (84). SAPH is classified as World Health Organization (WHO) group 5 due to its complex and multifactorial mechanisms (87). The optimal management for pulmonary hypertension in sarcoidosis is not well-defined. Along with treatment directed at active sarcoid inflammation (corticosteroid and steroid-sparing agents), therapy should focus on correcting hypoxia when present and managing comorbidities such as sleep apnea and cardiac dysfunction. Reduced diffusing capacity of the lung for carbon monoxide (DLCO) correlates with the severity of SAPH (88). Currently, the use of pulmonary hypertension specific therapy for the management of SAPH is controversial, and thus far pulmonary arterial hypertension specific therapies have not been approved (89, 90). Lung transplantation may also be considered in this high risk group. A reduced *D*LCO (<35% predicted) and a 6MWD of <300 m are associated with worse transplant-free survival. Oksana et al. identified preservation of FEV1/FVC ratio as an independent risk factor for worsened outcomes (91).

## Neurosarcoidosis and Its Potential Mimics-Aseptic Meningitis and Multiple Sclerosis

Any part of the nervous system may be involved in sarcoidosis. Neurological involvement occurs in 5–15% of patients and often precedes the diagnosis of sarcoidosis in up to 74%. Neurological Involvement most commonly affects cranial nerves (Cranial nerve VII and II are most common), the hypothalamus and the pituitary gland followed by the meninges, brainstem, spinal cord are less frequently involved (92–96).

Neurosarcoidosis can mimic other neurologic diseases including neoplasm (lymphoma, metastasis) (97), infectious etiologies (meningoencephalitis) (98) and other inflammatory diseases (angiitis/vasculitis, demyelinating disorders). Neurosarcoidosis can present as multiple supratentorial and/or infratentorial masses (35%) or solitary masses (15%) (99). The differential diagnosis includes gliomas, primary B cell lymphoma, metastatic disease, infarct, and demyelinating disease. Motor dysfunction is present in up to 50% of cases. Patients can demonstrate neuropsychiatric manifestations such as depression, psychoses, dementia, poor concentration, and hallucinations (100–102).

## Central Involvement

### Aseptic Meningitis Associated With Sarcoidosis

Cerebrospinal Fluid (CSF) findings in neurosarcoidosis may reveal elevated protein (50–70% of patients), elevated CSF pressure with a lymphocytic pleocytosis (57–72% of patients), and a reduced glucose level (up to 18% patients). None of these abnormalities are specific for neurosarcoidosis. Several studies evaluated the role of elevated CSF angiotensin-converting enzyme (ACE) level for diagnosing neurosarcoidosis (103, 104). The sensitivity of CSF ACE varies depending on the location of the central nervous system (CNS) involvement. For example, higher levels are rarely seen with spinal cord involvement (105). The ACE assay is not a specific test as it can be elevated in bacterial and viral encephalitis, neurosyphilis, malignant CNS tumors, Huntington's disease, multiple sclerosis and neuroleptic-treated schizophrenic patients (106). Steroid therapy can decrease the ACE level (103).

In suspected neurosarcoidosis associated aseptic meningitis, CSF should be sent for routine microbiological studies, fungal, and mycobacterium cultures, mycobacterium TB polymerase chain reaction (PCR). Cytology and flow cytometry should also be considered. Once the infection has been ruled out, corticosteroids can be initiated as first-line treatment. In sarcoidosis, both aseptic meningitis and isolated cranial nerve abnormalities usually respond to steroids. Corticosteroids can be started at 1 mg/kg or as a pulse of methylprednisolone (1,000 mg/day for 3 days). Steroids may be gradually reduced over the next 6–9 months. In steroid-refractory cases, methotrexate, azathioprine, cyclophosphamide, or mycophenolate should be considered. Radiotherapy has been reported in patients with refractory sarcoid meningitis but has been used infrequently (92, 107, 108).

### Multiple Sclerosis

One of the most challenging presentations is in patients, typically young women, with optic neuritis and finding a few demyelinating lesions on brain MRI. The clinical presentation of both neurosarcoidosis and multiple sclerosis (MS) include both these features. While over 90% of MS patients have oligoclonal bands on CSF analysis, oligoclonal bands can be seen in up to 25–50% of neurosarcoidosis patients making distinguishing sarcoidosis from multiple sclerosis more difficult (109–112). Neurosarcoidosis involvement on MRI demonstrates non-enhancing T2 and FLAIR white matter lesions often in a periventricular distribution mimicking the demyelinating lesions seen in MS (111, 113). Both MS and neurosarcoidosis can follow a relapsing or progressive course. A timely and correct diagnosis is essential because the misdiagnosis of neurosarcoidosis would prevent the patient from receiving highly effective MS therapies. Additionally, exposure to neurosarcoidosis specific therapies such as TNF-alpha antagonists may worsen MS (114, 115).

Table 4 illustrates the characteristic clinical and radiological features of Neurosarcoidosis, aseptic Meningitis and MS.

**Table 4:** Highlights the features of aseptic meningitis secondary to viral etiology and neuro-sarcoidosis associated meningitis.

	**Sarcoidosis**	**Multiple sclerosis**	**Aseptic meningitis associated with viral infection**
Laboratory	Elevated ACE level Abnormal calcium metabolism (hypercalciuria and/or hypercalcemia) (7, 12)	Non-specific laboratory findings	Enteroviruses most common, arboviruses, herpesviruses, influenza, mumps, HIV (109, 116, 117).
Eye involvement	Anterior uveitis Acute Bilateral optic neuritis (118, 119)	Unilateral optic neuritis, pain on eye movement, partial and mainly central visual blurring, normal disc or mild disc swelling	Ocular involvement not common.
Myelopathy	Can progress to spastic paraparesis MRI spine-meningeal and nerve root involvement (120)	Can progress to spastic paraparesis MRI spine-no meningeal enhancement has been reported.	Myelopathy is not common.
CSF findings	Lymphocytic Pleocytosis (57–72% patients) Elevated Protein (50–70% patients) Low glucose (18% patients) CSF ACE elevated 50% patients Oligoclonal bands Elevated CSF pressure finding of Kveim specific IgG in CSF (89, 121, 122)	Mild Lymphocytic Pleocytosis (<50/mm^3^) Protein may be elevated in 1/3 cases. Normal glucose and pressure (123, 124) Oligoclonal bands or elevated IgG index in CSF (125)	Mononuclear pleocytosis (may be neutrophilic in early stage) Protein concentration can be normal to elevated (typically 0.4–0.8 g/l). Normal to low glucose CSF PCR specific for virus (enterovirus, HSV, VZV) (109)
MRI findings	Variable contrast enhancement of the leptomeninges with a nodular or diffuse pattern, non-specific white matter lesions, and hydrocephalus.	Oval, asymmetric white matter lesions perpendicular to the ventricles (Dawson fingers) or in the periventricular, juxtacortical, infratentorial or spinal cord region, and having a diameter >6 mm are considered typical of MS (54, 126–129)	MRI demonstrates abnormalities specific to the viral etiological agent (107)
Treatment	Corticosteroids	Corticosteroids, DMDs	Supportive management Acyclovir in HSV

*CSF, Cerebrospinal fluid; MRI, Magnetic resonance imaging; PCR, Polymerase chain reaction; DMDs, Disease-modifying drugs; ACE, Angiotensin converting enzyme; MRI, magnetic resonance imaging*.

The diagnostic workup in a patient with a suspicion of neurosarcoidosis mimicking MS should include chest CT to search for lymphadenopathy and a full-body positron emission tomography (PET) scan to search for other organ involvement that supports the diagnosis of sarcoidosis, e.g., FDG uptake in thoracic lymph nodes, lung, spleen, and bone. PET scans can also direct tissue biopsy for histologic confirmation (118).

Diagnostic criteria proposed by Zajicek et al. categorizes neurosarcoidosis into definitive, probable, and possible neurosarcoidosis (129). Definite neurosarcoidosis is defined as clinical presentation suggestive of neurosarcoidosis after the exclusion of other possible diagnoses with the histological evidence of sarcoid in the nervous system. Probable neurosarcoidosis is defined as a suggestive clinical presentation of neurosarcoidosis in a patient with systemic sarcoid and after alternative diagnoses have been excluded. Possible neurosarcoidosis is defined as clinical presentation suggestive of neurosarcoidosis after alternative diagnoses have been excluded.

MRI is the preferred mode of imaging for diagnosis and follow up of neurosarcoidosis as it carries a high sensitivity. Leptomeningeal enhancement favors a diagnosis of sarcoidosis over neoplasm (123, 129–131). Enhancing parenchymal masses are an infrequent but important manifestation of sarcoidosis, but may also occur in primary lymphoma of the brain (97, 132). A CNS biopsy may be indicated when there is no extra-neurological disease, particularly if a lack of response to immunosuppressive treatment occurs. MRI can reveal accessible locations for biopsy, preferably from the leptomeninges (113).

Immunosuppression has been the mainstay of treatment for neurosarcoidosis with corticosteroids being the first-line. Other steroid-sparing therapeutic agents include methotrexate, mycophenolate mofetil, azathioprine, cyclosporine, cyclophosphamide, chlorambucil, pentoxifylline, hydroxychloroquine, thalidomide, infliximab, and adalimumab (133).

### Peripheral Involvement

Peripheral nervous system involvement includes mononeuropathy, mononeuritis multiplex, sensory, sensorimotor, and motor polyneuropathies. Symptoms may be acute, subacute, or chronic. An acute generalized demyelinating motor neuropathy similar to the Guillain-Barré syndrome also has been described (134). Small fiber neuropathy resulting in distal limb pain and impaired perception of temperature, hyperesthesia, and autonomic dysfunction have been recognized (135).

Steroid-induced remissions coupled with frequent relapses observed in patients with neurosarcoidosis can lead to a clinical picture that resembles an inflammatory demyelinating disease. Neurologic involvement may be more difficult to confirm as tissue biopsy may be impractical (136–138).

## Abnormal Calcium Metabolism

Harrel et al. first described hypercalcemia associated with sarcoidosis in 1939 and since then abnormal calcium metabolism is considered a key feature of sarcoidosis. Hypercalcemia in sarcoidosis is secondary to an uncontrolled synthesis of 1,25-dihydroxy vitamin D3 by macrophages present in granulomas. The increased 1,25 dihydroxyvitamin D3 leads to increased intestinal absorption of calcium that leads to increased resorption of calcium in the bone (139, 140). The most common manifestation of abnormal calcium metabolism is hypercalciuria which is prevalent in ~40–62% (119). Patients often provide a history of nephrolithiasis.

In the kidney, untreated hypercalcemia causes afferent arteriole vasoconstriction and inhibition of sodium-potassium ATPase causing a decrease in glomerular filtration rate and urinary sodium wasting, polyuria, and dehydration. Increased intracellular calcium overload and tubular obstruction by calcium precipitates may lead to tubular necrosis. Renal consequences of hypercalcemia and hypercalciuria are frequently reversible, however, long-standing hypercalciuria may lead to nephrocalcinosis and permanent changes (141, 142).

Once hypercalcemia is detected, serum albumin, ionized calcium, and 24-h urine collection for calcium excretion should be measured. In cases of progressive renal impairment, 24 h creatinine clearance, and abdominal ultrasound should also be performed to exclude nephrolithiasis or nephrocalcinosis. Primary hyperparathyroidism should routinely be ruled out. Histologic confirmation of sarcoidosis may be required to exclude lymphoma as it has the propensity to present with hypercalcemia associated with lymphadenopathy (143).

## Management

Table 5 highlights the laboratory diagnostics and management of hypercalcemia associated with sarcoidosis.

**Table 5:** Laboratory and radiological testing along with the management of hypercalcemia associated with sarcoidosis.

**Laboratory and radiological tests**	**Management**
24-h urine collection for calcium excretion. Parathyroid Hormone (PTH) and PTH-related peptide (PTHrP) Serum creatinine (and/or cystatin C) Serum calcium and albumin levels should be measured and the ionized calcium calculated. Renal Ultrasound to exclude nephrolithiasis Age appropriate malignancy work up (To rule out hypercalcemia secondary to malignancy)	**Hypercalciuria without stone formation**—observational approach; often requires no treatment. Close monitoring to prevent renal failure. Treatment options may include corticosteroids and/or diuretics. **Hypercalciuria with stone formation—**Bisphosphonates or corticosteroids should be considered; shockwave lithotripsy **Mild, asymptomatic** **Hypercalcemia—**Encourage fluid intake of >2 l per day and minimize their exposure to sunlight, avoid vitamin D and fish oil supplementation. **Moderate hypercalcemia—**May consider addition of corticosteroids and/or ketoconazole or hydroxychloroquine. **Severe hypercalcemia**—act immediately: rehydrate. Treatment options include corticosteroids, calcitonin, loop diuretics, and bisphosphonates (144)

Asymptomatic and mild hypercalcemia detected in a patient presenting with acute sarcoidosis requires no further assessment, and studies suggest that monitoring the response to corticosteroid therapy is an acceptable practice. Management is aimed to prevent long term renal and bone complications. Patients are advised to maintain a fluid intake of >2 l per day, minimize their exposure to sunlight, and avoid vitamin D. Severe hypercalcemia is fairly responsive to steroids. Corticosteroids achieve normocalcemia by inhibiting the enzyme 1-α-hydroxylase, reducing gastrointestinal calcium absorption, inhibiting osteoclast function, and decreasing the production of parathyroid hormone-related protein (PTHrP) by the macrophages (31, 144–147). Early recognition and treatment of abnormal calcium metabolism associated with sarcoidosis is important as long-standing untreated hypercalcemia can progress to irreversible renal failure. It is imperative to consider the implication of the use of corticosteroids in patients as it further increases the risk of developing glucocorticoid-induced osteoporosis (148).

## Sarcoid-Like Reactions (SLRs)

Sarcoid-like reaction (SLR) is defined as the presence of non-caseating epithelioid cell granuloma lesions of sarcoidosis without accompanying systemic symptoms. Sarcoid-like reactions are histologically indistinguishable from systemic sarcoidosis (149).

## Sarcoid-Like Reactions (SLRs) Associated With Malignancy

SLRs can be observed in patients with various malignancies. The lesions can be adjacent to the primary tumor, in the tumor itself or adjacent to the local draining lymph nodes (136, 150). SLRs occur in 4.4% of carcinomas, in 13.8% of patients with Hodgkin's disease, and in 7.3% of cases of non-Hodgkin lymphomas (151). Developing SLRs adjacent to the tumor sites may be due to a local reaction to tumor products or immunological response to an antigenic trigger. SLRs far from tumor sites may represent a host immune response to the soluble circulating tumor antigenic factors perhaps by acting as a type of auto-Kveim reagent (136, 152, 153).

Both SLRs and malignancy demonstrate increased uptake of 18F-fluorodeoxyglucose (FDG) on PET scan, which makes it difficult to distinguish the two. Though PET scans may be useful in selecting possible biopsy sites to search for malignancy, they have no role in differentiating between these two entities. There are no specific markers or radiographic patterns to distinguish SLRs from systemic sarcoidosis or from malignancy (153–156). SLRs may have an improved prognosis as a few studies have reported that SLR is self-limited (151, 157, 158). The occurrence of malignancy and sarcoidosis concomitantly is unusual but a few cases have been reported (158, 159). The development of hilar and/or mediastinal lymphadenopathies in patients with a history of malignancy should prompt considering SLRs. Biopsies of affected tissue are often required to rule out cancer recurrence.

## Drug-Induced Sarcoidosis-Like Reactions (DISRs)

Drug-induced sarcoidosis-like reactions (DISR) can be defined as a granulomatous tissue reaction, indistinguishable from sarcoidosis, that occurs at the same time as initiation of a potential offending drug (160). DISR can be associated with typical sarcoid-like manifestation including bilateral hilar lymphadenopathy, uveitis, hypercalcemia, cutaneous lesions, elevated serum ACE levels, and FDG uptake on PET scans (161–165). A variety of drugs have been implicated as causing DISRs including immune checkpoint inhibitors, tumor necrosis factor (TNF)-α antagonists, interferons (IFN), and antiretroviral drugs. It is unclear if the etiology of DISR is that of a sarcoid-like reaction *per se* or a result of an impaired immune system leading to the development of sarcoidosis while on drug therapy (160).

Although TNF-α is known to play an important role in the formation and stabilization of sarcoid granulomas, surprisingly DISRs have been reported with the use of TNF-α antagonists therapy. Most commonly reported with etanercept, DISRs occur with any of the TNF-α antagonists. The average time to occurrence of DISRs is 24 months after drug initiation with almost 60% of patients requiring treatment (166). Treatment requirements range from 0 to 59.2% in reported cases of DISR, depending on the class of drug implicated (Table 6).

**Table 6:** Highlights the drugs commonly associated with DISR.

	**Drug class**	**Percentage of patients requiring anti-sarcoidosis treatment**	**References**
1.	Interferon (interferon alpha and interferon beta)	42.9% (157)	(164, 166–168)
2.	Tumor necrosis factor-α antagonist	59.5% (157)	(169, 170)
3.	BRAF inhibitor	0%	(171, 172)
4.	Interleukin-1 receptor antagonist	0%	(173)
5.	Immune checkpoint inhibitors (nivolumab, pembrolizumab, ipilimumab)	57.2% (157)	(174, 175)
6.	Highly active antiretroviral therapy (HAART)	41.1% (157)	(176–178)
7.	Botulinum neurotoxin A	0% (157)	(179)

DISRs may mimic other clinical manifestations of sarcoidosis such as infections, drug and autoimmune reactions, and neoplasms. In select cases, DISR can resolve with discontinuation of the offending drug (160, 167). DISRs that are not associated with significant symptoms, organ dysfunction, or quality of life impairment, do not often require treatment. In cases where treatment is required, standard sarcoidosis treatment regimens can be utilized (160).

## Implant and Device-Induced Sarcoid-Like Reactions

SLRs have been reported after joint replacement surgery and after silicone breast implant placement (180, 181). These reactions are postulated to be due to an autoimmune inflammatory syndrome induced by adjuvants (ASIA). The pathogenesis of the development of ASIA is not clearly defined but one proposed mechanism involves adjuvants (i.e., silicone, mineral oil, hyaluronic acid) chronically stimulating the immune pathway and preventing antigens from being degraded which can then enhance the antigen exposure to the antigen-presenting cells (APC) (182, 183). Definitive treatment requires the removal of the prosthetic implant.

## Cardiac Involvement With Sarcoidosis and Differentiating It From Potential Mimics

In 1929, Bernstein was the first to recognize cardiac involvement in sarcoidosis. Granulomas in cardiac sarcoidosis (CS) most often involve the conduction system and can potentially involve any part of the heart including the pericardium and myocardium (184). Depending upon the location and extent of granulomatous inflammation, clinical manifestation range from asymptomatic conduction abnormalities to fatal heart block and ventricular arrhythmia and congestive heart failure (CHF). In CS, CHF is the second most frequent cause of death after sudden cardiac death. Pericardial effusions are infrequent in CS and rarely progress to cardiac tamponade (184, 185).

A timely diagnosis of CS is often difficult as it may present with clinical features and radiographic findings overlapping with other cardiac disorders. Plain chest radiographs may appear normal. Some of the common mimickers of CS cardiomyopathy include amyloidosis and other infiltrative diseases, non-ischemic and ischemic cardiomyopathy, right ventricle infarction, lymphocytic myocarditis, connective tissue diseases associated cardiomyopathy, vasculitis (Takayasu arteritis and Wegener granulomatosis), Chagas disease, hypertrophic cardiomyopathy, and other infectious causes associated with cardiomyopathy (e.g., rheumatic fever, syphilis, fungal infections, and TB) (186–192).

Echocardiographic abnormalities, present in up to 24–77% of CS patients, range from non-specific findings to more sarcoid associated findings such as thinning of the basal anterior septum, regional wall aneurysm, diastolic dysfunction, or motion wall abnormalities in a non-coronary artery distribution (193). Impaired regional peak systolic longitudinal strain on strain echocardiography has been described and associated with cardiovascular events (194).

Thallium 201 scintigraphy can be used to help distinguish the ischemic disease from sarcoidosis or other infiltrative diseases. The reverse distribution phenomenon on rest and exercise thallium described as defects detected in the resting phase during thallium scanning that disappears or decreases in size during exercise or after dipyridamole infusion (195). Cardiac PET scanning may be combined with MRI to further define CS involvement. The sensitivities of cardiac PET and MRI with contrast are about the same for detecting myocardial involvement. Late gadolinium enhancement (LGE) on MRI generally cannot distinguish scar from inflammation while FDG uptake implies active inflammation (196, 197). Cardiac PET scanning is particularly useful in patients unable to undergo cardiac MRI because of the presence of implantable cardiac devices or renal failure where gadolinium is contraindicated.

Infrequently, granulomatous infiltration of the myocardium can result in myocardial thickening which can morphologically mimic hypertrophic cardiomyopathy but unlike CS, which is characterized by inflammation and edema, T2-weighted MRI features not typically seen in hypertrophic cardiomyopathy. Areas of Late gadolinium- enhancement (LGE) in CS are more likely to be patchy and mid myocardial.

For cardiac amyloid which is a consideration for unexplained infiltrative disease, both 99mTcpyrophosphate scanning and tissue biopsy (fat pad) showing apple-green birefringence at polarizing light microscopy are distinguishing features (198–200). In patients with CS and extracardiac involvement lymph node or lung biopsy is typically targeted but in cases of negative extracardiac biopsy or isolated CS, MRI, or PET directed endomyocardial biopsy may be required. Electro-anatomic mapping can also be used to choose cardiac sites for biopsy (201, 202).

CS can be managed with corticosteroids and when necessary inotropic, vasodilator, and antiarrhythmic medications. Automatic Implantable Cardioverter Defibrillator (AICD) or cardiac pacing may have a role. Cardiac transplantation although rare can be considered in younger patients with advanced end-stage cardiac failure. No difference in outcome has been reported when transplants are done for CS (203–206).

## Common Variable Immunodeficiency Syndrome (CVID)

Common variable immunodeficiency syndrome may be complicated by granulomatous lymphocytic interstitial lung disease (GLILD) (205). The presence of sarcoidosis like lesions associated with hypogammaglobulinemia should suggest a diagnosis of CVID as up to 8–22% of patients with CVID can present with GILD (207–209). In a retrospective analysis more than two-thirds of CVID patients with radiographic evidence of interstitial lung disease had GLILD. GLILD was also frequently reported with dyspnea and splenomegaly. These patients can present with a clinical picture that mimics sarcoidosis and misdiagnosis can delay the treatment for CVID (IVIG), which in the presence of GLILD portends a poorer prognosis. Steroid therapy can further worsen CVID (208, 210–212).

Key clinical features that should alert one to the diagnosis of CVID include a history of recurrent infections (213) and a concurrent diagnosis of autoimmune diseases. Concurrent autoimmune disease occurs much less frequently in sarcoidosis than CVID. Sarcoidosis is more commonly associated with uveitis and skin disease whereas CVID patients more frequently have autoimmune cytopenias. Comparing GLID to sarcoidosis, chest CT scans had more air bronchogram, halo signs, and smooth bordered nodules in the CVID group. Bronchoalveolar lavage (BAL) revealed lower T-cell CD4: CD8 as well in the CVID group (214, 215).

With the aid of B cell immunophenotyping, granulomas associated with CVID reveal a severe reduction in switched memory B cells and a high level of CD21 low B cells. Immunoglobulin perfusions are considered the treatment of choice for CVID (216). Measurement of serum immunoglobulins should be considered when a history of recurrent infections is given and should be considered in any patient with granulomatous disease, even if the clinical and radiological presentation appears typical for sarcoidosis (217).

## Conclusion

As illustrated by the numerous diseases that sarcoidosis can imitate, sarcoidosis deserves the reputation of “The Great Mimicker.” The purpose of this review the most common diseases that should be considered when sarcoidosis enters the differential diagnoses. Aside from the time lost for adequate treatment of the disease present, both using immunosuppressive drugs in a state that appears to be sarcoidosis or conversely failing to adequately treat sarcoidosis with its proper treatment regimen will lead to adverse outcomes. The authors herein described common pitfalls in the diagnostic process and how to avoid them in order to provide, to the best of our ability, an accurate diagnosis for a condition that continues to baffle, elude, and mislead clinicians.

## Author Contributions

All authors contributed equally in data collection, interpretation, paper writing, editing, and review.

## Conflict of Interest

The authors declare that the research was conducted in the absence of any commercial or financial relationships that could be construed as a potential conflict of interest.
